# Systematic evaluation of RNA quality, microarray data reliability and pathway analysis in fresh, fresh frozen and formalin-fixed paraffin-embedded tissue samples

**DOI:** 10.1038/s41598-018-24781-6

**Published:** 2018-04-20

**Authors:** Isabella Wimmer, Anna R. Tröscher, Florian Brunner, Stephen J. Rubino, Christian G. Bien, Howard L. Weiner, Hans Lassmann, Jan Bauer

**Affiliations:** 1 Department of Neuroimmunology, Center for Brain Research, Medical University of Vienna, Vienna, Austria; 2 Ann Romney Center for Neurological Diseases, Brigham and Women’s Hospital, Harvard Medical School, Boston, USA; 3 Epilepsy Center Bethel, Krankenhaus Mara, Bielefeld, Germany

## Abstract

Formalin-fixed paraffin-embedded (FFPE) tissues are valuable resources commonly used in pathology. However, formalin fixation modifies nucleic acids challenging the isolation of high-quality RNA for genetic profiling. Here, we assessed feasibility and reliability of microarray studies analysing transcriptome data from fresh, fresh-frozen (FF) and FFPE tissues. We show that reproducible microarray data can be generated from only 2 ng FFPE-derived RNA. For RNA quality assessment, fragment size distribution (DV200) and qPCR proved most suitable. During RNA isolation, extending tissue lysis time to 10 hours reduced high-molecular-weight species, while additional incubation at 70 °C markedly increased RNA yields. Since FF- and FFPE-derived microarrays constitute different data entities, we used indirect measures to investigate gene signal variation and relative gene expression. Whole-genome analyses revealed high concordance rates, while reviewing on single-genes basis showed higher data variation in FFPE than FF arrays. Using an experimental model, gene set enrichment analysis (GSEA) of FFPE-derived microarrays and fresh tissue-derived RNA-Seq datasets yielded similarly affected pathways confirming the applicability of FFPE tissue in global gene expression analysis. Our study provides a workflow comprising RNA isolation, quality assessment and microarray profiling using minimal RNA input, thus enabling hypothesis-generating pathway analyses from limited amounts of precious, pathologically significant FFPE tissues.

## Introduction

Pathological archives contain vast amounts of formalin-fixed paraffin-embedded (FFPE) specimen from routinely collected biopsies and autopsy tissue as well as precious material derived from rare disease cases or such with exceptional pathogenesis^1,2^. Contrary to experimental animal studies, which are often feasible with fresh frozen (FF) material, research relying on human tissues is predominantly dependent on FFPE material.

FFPE tissue blocks are long-lasting and easy to archive. Formaldehyde fixation (using formalin or paraformaldehyde) excellently preserves cellular structures and tissue morphologies by introducing inter- and intramolecular cross-links between side chains of amino acids^3,4^, thereby maintaining secondary and tertiary structures of proteins^5,6^. However, the fixatives chemically modify nucleic acids as well^7,8^. The addition of methylol groups to bases leads to the formation of methylene bridges between amino groups similarly as for proteins. Furthermore, hydrolysis of N-glycosylic bonds creates abasic sites, while hydrolysis of phosphodiester bonds introduces breaking points in the sugar-phosphate backbone resulting in nucleic acid fragmentation. Moreover, proteins and nucleic acids are cross-linked to each other upon fixation^9^.

In addition to fixation methods^8,10^, studies have identified tissue fixation time, specimen size, and tissue storage conditions as important factors influencing RNA quality^11,12^. Moreover, the choice of tissue lysis and isolation kits influences yield and quality of isolated RNA fragments^13–16^. However, available information on tissue processing of archival material is often incomplete. Thus, it is essential to integrate appropriate control steps to monitor RNA quality and functionality.

Microarray technology and bioinformatics tools have advanced tremendously in recent years and their use has been extended to a broad spectrum of biologically relevant research questions. Together with advanced transcription kits specifically designed for low input derived from microdissected tissue, this opens up greater opportunities for basic and clinically-related research.

In the present study, we assessed the usability of low quantities of FFPE tissue-derived RNA for microarray studies in comparison with FF tissue- and fresh tissue-derived samples using brain tissue derived from humans, rats and mice. Based on the results, we are able to provide an experimental workflow of RNA isolation, quality determination and microarray preparation that can be applied to as little as 2 ng total RNA input. Our findings pave the way for unlocking the transcriptome of countless FFPE material, which can be found in archives and tissue banks and is of great interest especially for human molecular pathology.

## Results

**Figure 1:**
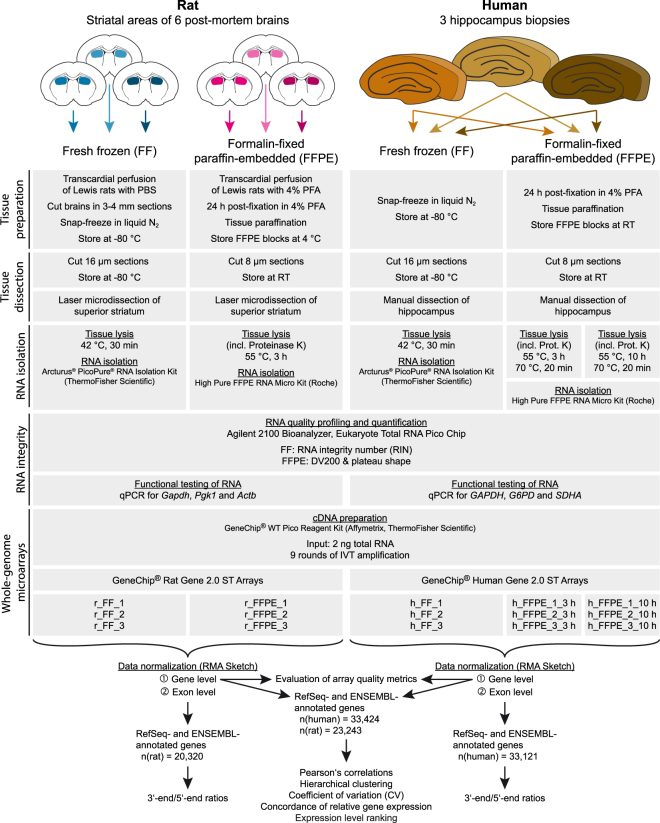
Schematic overview of the study.

To test for the effect of formalin fixation on microarray data reliability and reproducibility, we performed a side-by-side analysis of paired fresh frozen (FF) and formalin-fixed paraffin-embedded (FFPE) material derived from striatal and hippocampal tissue from rats (r_FF, r_FFPE) and humans (h_FF, h_FFPE_3 h and h_FFPE_10 h), respectively (Fig. 1).

### Size distribution of RNA fragments combined with qPCR is suitable for quality assessment of FFPE-derived RNA

**Figure 2:**
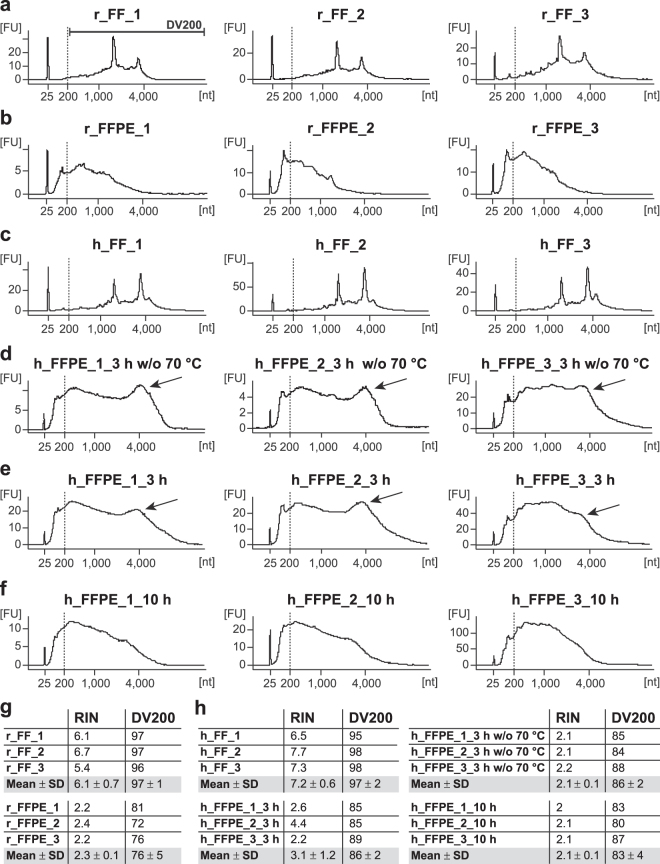
Agilent Bioanalyzer electropherograms as suitable basis for RNA quality assessment. **(a-f)** Isolated total RNA was loaded on Agilent RNA Pico Chips, which were run in an Agilent 2100 Bioanalyzer. RNA profiles of microdissected rat (**a**) and human (**c**) FF samples show clearly distinguishable 18 S and 28 S rRNA peaks, which are absent in FFPE samples (**b**,**d-f**). Instead, we examined the shape of the plateau and used the DV200 to judge RNA quality of FFPE samples. In human FFPE samples, prolongation of tissue lysis led to a decrease of high-molecular-weight species, since the peak with an average size of 3,000–4,000 nucleotides (**d,e**; arrows) is no longer present after 10 hours incubation (**f**). Implementation of a 70 °C incubation step after tissue lysis (**e**,**f**) favoured cross-link reduction as well, noticeable by a reduction of the peak on the right-hand side as compared with corresponding untreated samples (**d**). FU, fluorescent units; nt, nucleotides; DV200, percentage of RNA fragments >200 nucleotides. **(g**,**h)** Overview of RIN and DV200 parameters of rat (**g**) and human (h) FF and FFPE samples to monitor RNA quality. Increasing the lysis time of human tissues (h) from 3 to 10 hours did not significantly affect DV200 values (unpaired two-tailed Student’s t-test; *p* = 0.2840, t(4) = 1.236), nor did the additional incubation step at 70 °C (*p* = 0.7292, t(4) = 0.3714). RIN, RNA integrity number; DV200, percentage of RNA fragments >200 nucleotides.

**Figure 3:**
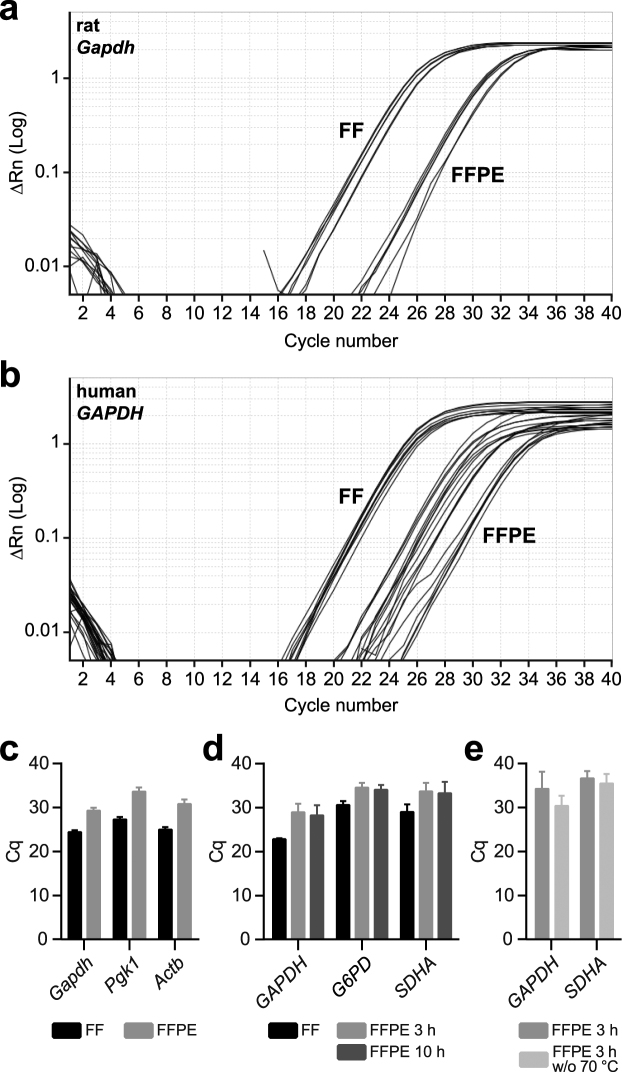
Functional RNA quality control by qPCR. **(a**,**b)** Representative amplification plots for the reference gene *Gapdh* (data for 5′-end primer pairs) using either rat (**a**) or human (**b**) templates derived from FF or FFPE tissue. **(c**,**d)** Cq values for reference genes using either rat (**c**) or human (**d**) templates isolated from FF and FFPE tissues. For each gene and template, qPCR with either 3′-end or 5′-end primer sets was run for 40 cycles in duplicates (rat) or triplicates (human). Cq values are depicted as geometric mean ± SD and represent the averages from 3′-end and 5′-end qPCRs. Cq values of h_FFPE_3 h and h_FFPE_10 h samples did not differ significantly (unpaired two-tailed Student’s t-tests; *GADPH*: *p* = 0.6935, t(4) = 0.4239; *G6PD*: *p* = 0.6398, t(4) = 0.5055; *SDHA*: *p* = 0.8427, t(4) = 0.2117). (**e**) Cq values for reference genes using human FFPE-derived cDNA. FFPE_3 h samples included an additional incubation step at 70 °C, which was omitted for FFPE_3 h w/o 70 °C samples. For qPCR, solely primer sets annealing to the 3′-end of the mRNA were employed. Cq values are depicted as geometric means ± SD. Cq values of h_FFPE_3 h and h_FFPE_3 h w/o 70 °C samples did not differ significantly (unpaired two-tailed Student’s t-tests; *GADPH*: *p* = 0.2075, t(4) = 1.502; *SDHA*: *p* = 0.5847, t(3) = 0.6104). Rn, fluorescence of SYBR Green in relation to the passive reference dye ROX; Δ Rn, Rn minus baseline; Cq, quantification cycle; Gapdh, glyceraldehyde-3-phosphate dehydrogenase; Pgk1, phosphoglycerate kinase 1; Actb, actin beta; G6pd, Glucose-6-phosphate dehydrogenase; Sdha, succinate dehydrogenase complex flavoprotein subunit A.

Total RNA from FF- and FFPE-derived samples was loaded on Agilent RNA Pico Chips to determine RNA quantity and integrity. Rat and human FF samples showed clearly distinguishable 18 S and 28 S rRNA peaks (Fig. 2a,c) and RNA integrity numbers (RINs) were calculated to an average of 6.1 and 7.2, respectively (Fig. 2g,h). For FFPE samples, rRNA peaks were not identifiable (Fig. 2b,d–f) and RINs were reduced to a level considered as indicator for degraded RNA (Fig. 2g,h). Thus, as alternative quality measures for FFPE-derived RNA, we evaluated Bioanalyzer electropherograms and calculated DV200 values (percentage of RNA fragments with a length >200 nucleotides), reliable quality determinants for FFPE-derived RNA^17^. RNA electropherograms represent the size distribution of RNA fragments. Thus, for FFPE-derived samples, a long plateau is favourable, indicating a high number of long RNA fragments. However, after 3 hours of lysis of human tissue, a well delimited peak with an average size of 3,000–4,000 nucleotides was present (arrows in Fig. 2d,e) suggestive of remaining cross-linked nucleic acids^18,19^. When we increased the tissue lysis time to 10 hours, the high-molecular-weight species were decreased, putatively indicating more efficient retrieval of cross-linked RNA (absence of peak; Fig. 2f), without significantly affecting DV200 values (Fig. 2h). To increase RNA yields of human FFPE-derived samples, we included an incubation step at 70 °C (Fig. 1) resulting in almost 2.5-times higher average amounts of RNA for similar tissue inputs (h_FFPE_3 h w/o 70 °C: 37 ng; h_FFPE_3 h: 83 ng). DV200 values were not significantly affected by this advancement (Fig. 2h). Generally, all our FF and FFPE samples fell into the high-quality category (DV200 > 70%) according to the Illumina scale^17^. Moreover, they passed functional integrity testing by yielding sufficient amounts of PCR products for selected reference genes (Fig. 3). To keep this transcript analysis as uniform and unbiased as possible, we used a mix of oligo(dT) and random hexamer primers for reverse transcription and primer sets annealing near the 5′- and 3′-end of the mRNA for qPCR (Table 1). Although the same amounts of input were used for reverse transcription, FF samples resulted in smaller Cq values than FFPE samples for the tested reference genes (rat: *Gapdh*, *Pgk1* and *Actb*; human: *GAPDH*, *G6PD* and *SDHA*) indicating more PCR products (Fig. 3c,d). Representative amplification plots illustrate the clear separation of FF from FFPE samples (Fig. 3a,b). Although Bioanalyzer profiles differed between h_FFPE_3 h and h_FFPE_10 h, no significant differences in the functional quality of the isolated RNA were observed (Fig. 3d). Also the additional incubation at 70 °C step did not influence any RNA quality-related parameters (Fig. 3e). Altogether, the combination of visual inspection of the shape of Bioanalyzer electropherograms, calculation of the DV200 and functional testing via qPCR proved as ideal for judging the suitability of FFPE-derived RNA for downstream applications.

**Table 1:** Details on qPCR primers.

Gene symbol	Species		Primer sequence	Product size	Primer efficiency
Gapdh	rat	forward (5′ end)	5′-TGTGAAGCTCATTTCCTGGTA-3′	88 bp	105%
		reverse (5′ end)	5′-TTACTCCTTGGAGGCCATGT-3′		
		forward (3′ end)	5′-GTCGGTGTGAACGGATTTG-3′	85 bp	113%
		reverse (3′ end)	5′-TGATGGCAACAATGTCCACT-3′		
Pgk1	rat	forward (5′ end)	5′-AAAGGATCAAGGCTGCTGTC-3′	74 bp	110%
		reverse (5′ end)	5′-GCTCATAAGCACAACCGACT-3′		
		forward (3′ end)	5′-GCCTGTGAAAGGAAGTGAGC-3′	98 bp	113%
		reverse (3′ end)	5′-CCGTTATTTCCATGCTGTCA-3′		
Actb	rat	forward (5′ end)	5′-AGGCCAACCGTGAAAAGATG-3′	101 bp	112%
		reverse (5′ end)	5′-ACCAGAGGCATACAGGGACAA-3′		
		forward (3′ end)	5′-GATTACTGCCCTGGCTCCTA-3′	90 bp	111%
		reverse (3′ end)	5′-AGGATAGAGCCACCAATCCA-3′		
Gfap	rat	Rn_Gfap_1_SG QuantiTect Primer Assay (QT00195517, Qiagen)			
Atrn	rat	forward	5′-GTTCGGAGGGAACACACACA-3′	98 bp	105%
		reverse	5′-ACCATCGGTCACAAGCAATG-3′		
Mbp	rat	forward	5′-AGAACATTGTGACACCTCGTA-3′	110 bp	123%
		reverse	5′-GTAGCCAAATCCTGGCTTCT-3′		
Mag	rat	forward	5′-CAGACCATCCAACCTTCTGTA-3′	108 bp	118%
		reverse	5′-AGTTGGGAATGTCTCCTGATT-3′		
Cx3cr1	rat	forward	5′-TGCTCAGGACCTCACCAT-3′	101 bp	117%
		reverse	5′-CCACGATGTCACCCAAATAA-3′		
GAPDH	human	forward (5′ end)	5′-ATATTGTTGCCATCAATGACCC-3′	121 bp	92%
		reverse (5′ end)	5′-ATGACAAGCTTCCCGTTCTC-3′		
		forward (3′ end)	5′-CATTTCCTGGTATGACAACGA-3′	120 bp	95%
		reverse (3′ end)	5′-CTTCCTCTTGTGCTCTTGCT-3′		
G6PD	human	forward (5′ end)	5′-GACATCCGCAAACAGAGTG-3′	146 bp	87%
		reverse (5′ end)	5′-GCATTCATGTGGCTGTTGA-3′		
		forward (3′ end)	5′-ACCTACGGCAACAGATACA-3′	141 bp	97%
		reverse (3′ end)	5′-CAGCAGTGGGGTGAAAATAC-3′		
SDHA	human	forward (5′ end)	5′-GGCAGGGTTTAATACAGCAT-3′	126 bp	95%
		reverse (5′ end)	5′-TAGAAATGCCACCTCCAGTT-3′		
		forward (3′ end)	5′-AAGGTGCGGATTGATGAGTA-3′	150 bp	97%
		reverse (3′ end)	5′-TTTGTCGATCACGGGTCTAT-3′		
ACTB	human	forward	5′-GCACCACACCTTCTACAATGAG-3′	123 bp	90%
		reverse	5′-AAGGTCTCAAACATGATCTGGG-3′		
PGK1	human	forward	5′-TGCTGGACAAAGTCAATGAG-3′	109 bp	93%
		reverse	5′-GCTCCCTCTTCATCAAACAG-3′		
CASP1	human	forward	5′-TGATGCTATTAAGAAAGCCCAC-3′	72 bp	103%
		reverse	5′-GAAACATTATCTGGTGTGGAAGAG-3′		
IL1R1	human	forward	5′-AGACAAGGCCTTCTCCAAGA-3′	102 bp	121%
		reverse	5′-CGTTCCTTGCATTTATCAGCCTC-3′		
AIF1	human	forward	5′-TGAGCCAAACCAGGGATTTA-3′	99 bp	93%
		reverse	5′-CGTCTAGGAATTGCTTGTTGA-3′		
IL18	human	forward	5′-CCTTTAAGGAAATGAATCCTCCTG-3′	95 bp	117%
		reverse	5′-CATCTTATTATCATGTCCTGGGAC-3′		
TLR3	human	forward	5′-TCATCCAACAGAATCATGAGAC-3′	115 bp	85%
		reverse	5′-CTTCATGGCTAACAGTGCAC-3′		
HMBS	human	forward	5′-AGCCCAAAGATGAGAGTGAT-3′	95 bp	103%
		reverse	5′-TACGAGGCTTTCAATGTTGC-3′		
CX3CR1	human	forward	5′-CCAGTGGGGCCTTCA-3′	100 bp	81%
		reverse	5′-ACCACGATGTCCCCAATATAA-3′		
ITGAM	human	forward	5′-TCAGTGGTGCCTGCAA-3′	100 bp	104%
		reverse	5′-GACATAAGGTCAAGGCTGTTA-3′		

Gapdh, glyceraldehyde-3-phosphate dehydrogenase; Pgk1, phosphoglycerate kinase 1; Actb, actin beta; Gfap, glial fibrillary acidic protein; Atrn, attractin; Mbp, myelin basic protein; Mag, myelin-associated glycoprotein; Cx3cr1, chemokine (C-X3-C motif) receptor 1; G6pd, glucose-6-phosphate dehydrogenase; Sdha, succinate dehydrogenase complex flavoprotein subunit A; Casp1, caspase 1; Il1r1, interleukin 1 receptor, type I; Aif1, allograft inflammatory factor 1; Il18, interleukin 18; Tlr3, toll-like receptor 3; Hmbs, hydroxymethylbilane synthase; Itgam, integrin alpha M.

### Whole-genome microarrays can be reliably performed with low inputs of FF- or FFPE-derived RNA

**Figure 4:**
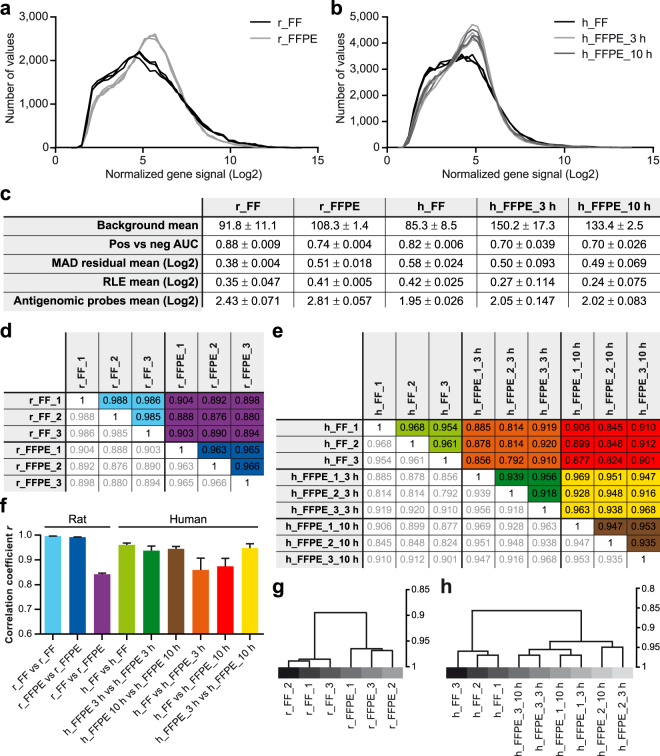
Microarray performance metrics. **(a**,**b)** Frequency distribution of normalized microarray data. Histograms depict rat (**a**) and human (**b**) gene signals (rat: n = 36,685 genes; human: n = 53,617 genes) after data normalization and transformation (Log2). Each microarray dataset is represented by a distinct line. (**c**) Quality assessment metrics for each experimental group. Performance parameters before (background mean) and after (all other parameters) data normalization were monitored via Affymetrix Expression Console^TM^ software. Reported values are mean ± SD (n per experimental group = 3 microarrays). AUC, area under curve; MAD, mean absolute deviation; RLE, relative log expression **(d**,**e)** Correlation matrices of Pearson’s correlation coefficients. Intra- and inter-experimental group correlations were calculated based on Log2-transformed rat (**d**; n = 23,243 genes) and human (**e**; n = 33,424 genes) data. All correlations were highly significant (*p* < 0.001). Grey values are replicates of colour-coded correlation. **(f)** Average Pearson’s correlation coefficients of intra- and inter-experimental group comparisons. Colour codes are matched between panels d-f. Data are represented as mean ± SD. Intra-experimental group correlations: n = 3; inter-experimental group correlations: n = 9 **(g**,**h)** Dendrograms illustrating unsupervised hierarchical clustering (Pearson’s correlation; average linkage) of rat (**g**) and human (**h**) microarrays (rat: n = 23,243 genes; human: n = 33,424 genes).

For both FF and FFPE samples, the input for each Affymetrix GeneChip^TM^ was only 2 ng of total RNA. We aggregated and normalized the raw data from FF and FFPE microarrays of each species to gene level using RMA-Sketch algorithms. Histograms of normalized gene signals illustrate FF and FFPE arrays as separate data entities with FFPE datasets showing a more pointy distribution than FF datasets (Fig. 4a,b). Quality assessment metrics differed between FF and FFPE arrays as well as between species (Fig. 4c); nevertheless, all metrics were in optimal ranges. Background means calculated prior to normalization were higher in FFPE than in FF arrays. However, the average detection rate of true positive over true negative probe sets (pos vs neg area under curve; AUC) for FFPE samples was in the range of optimal signal separation (>0.5) similarly as for FF samples. Likewise, both the mean absolute deviation (MAD) residual mean and the mean absolute relative log expression (RLE) differed between FF and FFPE samples; however, no outlying datasets were detectable. Signal intensities of antigenomic probes, which can be used as estimates of post-normalization background noise, were slightly higher in FFPE microarrays than in FF arrays. Additionally to the joint normalization (for each species: FF and FFPE combined), we assessed quality metrics for separately normalized datasets (for each species: FF only and FFPE only) as well. No obvious differences in performance parameters could be detected (data not shown). For this reason and since the employed normalization algorithms are more robust with larger sample sizes, we based all further analyses on collectively normalized datasets, from which RefSeq- or ENSEMBL-annotated genes were included.

### High intra- and inter-experimental group correlation of FF and FFPE whole-genome microarrays

Using Pearson’s correlation, we compared the individual array datasets with each other (intra- and inter-experimental group comparisons; Fig. 4d–f). For rat and human samples, the biological replicates within each experimental group displayed high correlation coefficients of at least 0.93. For human microarrays, the comparison of the two different lysis time conditions showed a high correlation, not only between the biological pairs themselves but also between all replicates. Inter-experimental group comparisons between FF and FFPE arrays yielded slightly lower but still remarkably high correlation coefficients. Using unsupervised hierarchical clustering, the biological triplicates within the experimental groups r_ FF, r_FFPE and h_FF clustered closely together (Fig. 4g). In the case of h_FFPE arrays, the paired 3 h and 10 h arrays of each case were similar, while the distances between the three different human cases were slightly higher (Fig. 4h). Thus, cluster analysis strengthened correlation results indicating a close relation of h_FFPE_3 h and h_FFPE_10 h datasets as well as a clear separation but still tight relation between FF and FFPE arrays.

### High reproducibility and concordant gene behaviour in FF and FFPE microarrays

**Figure 5:**
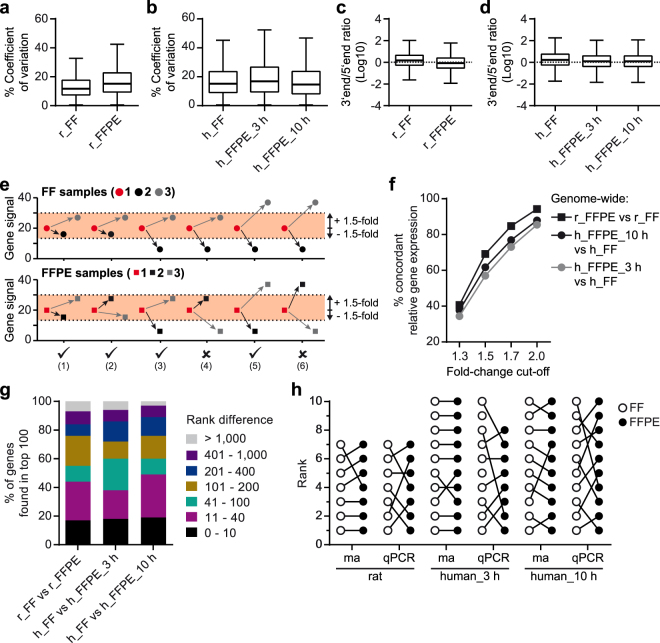
Microarray reproducibility and concordance between FF and FFPE datasets. **(a**,**b)** Coefficients of variation (CV, in percent) calculated for rat (**a**) and human (**b**) microarrays (rat: n = 23,243 genes; human: 32,914 to 33,251 genes). Increasing the lysis time (**b**) significantly affected %CV, but the effect size was minimal (Student’s t-test; p < 0.0001, t(65,926) = 19.15, d = 0.149). **(c**,**d)** Log10-transformed 3′-end/5′-end ratios of FF and FFPE microarrays based on rat (**c**) and human (**d**) samples normalized to exon level (rat: n = 15,325 genes; human: n = 24,776 genes). A positive ratio indicates a higher contribution of the 3′-end probe set, while a negative ratio implies a stronger signal at the 5′-end. **(e)** Graphical representation of the principle of concordance calculation. For each gene in each experimental group, ratios between the biological replicates (red, black and grey dots and squares) were calculated. Concordant relative gene expression was assumed if the ratios either lay within a defined fold-change cut-off (e.g. ± 1.5 as indicated by coloured areas; exemplified by conditions 1 and 2) or showed a comparable up- or down-regulation behaviour as depicted by conditions (3) and (5). (**f**) Rate of concordant relative gene expression (in percent) for four different fold-change cut-offs. Data points indicate mean concordance rates calculated for each gene (rat: 23,243 genes; human: 33,424 genes) with each of the biological triplicates serving as basis once. (**g**) Genes within each experimental group were sorted and ranked according to their expression rate. Each rank of the 100 highest expressed genes in FF arrays was compared with the corresponding rank in FFPE datasets. The resulting rank differences were categorized as shown. Rat and human datasets comprised 23,243 and 33,424 genes, respectively. (**h**) Comparison of ranked gene expression signals. For selected genes (n(rat) = 7; n(human) = 10), expression values determined by microarray and qPCR were sorted within each experimental group according to transcript abundance and assigned ascending ranks (highest expressed gene = rank 1). For each gene, ranks in FF datasets were compared with the corresponding ranks in FFPE datasets (indicated by lines). ma, microarray.

We determined the consistency of microarray data by several different means. First, the reproducibility of gene expression signals within FF and FFPE datasets was measured by the coefficient of variation (CV), the ratio between the standard deviation and the mean. For each experimental group, we calculated the %CV for every RefSeq- or ENSEMBL-annotated gene (Fig. 5a,b). The median CV values of rat and human arrays were in a consistently low range indicative of high data repeatability. For human arrays, prolongation of tissue lysis time from 3 hours to 10 hours slightly decreased the median CV suggestive of an improvement in data reproducibility. However, effect size calculation indicated an only minor impact on the overall outcome (Fig. 5b).

In the second analysis, we assessed the extent of concordant gene expression between FF and FFPE microarrays. Since FF and FFPE arrays constituted separate data entities (Fig. 4a,b), absolute gene signals could not be directly compared between the different experimental groups. Instead, relative expression changes between triplicates of each group are evaluable and should stay relatively constant between FF and FFPE datasets. To test this, ratios between the biological replicates within each experimental group (represented by arrows in Fig. 5e) were calculated for each RefSeq- or ENSEMBL-annotated gene. Concordant relative gene expression was attested if the ratios of the FF and the FFPE group were either within a certain fold-change cut-off range (conditions (1) and (2) in Fig. 5e) or showed comparable up- or down-regulation (conditions (3) and (5) in Fig. 5e). We tested concordant relative gene expression for four different fold-change cut-offs: 1.3, 1.5, 1.7 and 2.0. Concordance levels steadily increased with augmenting fold-change cut-offs from 40% at a fold-change cut-off of ±1.3 to 90% at a fold-change cut-off of ±2 (Fig. 5f).

We further pursued the question whether the analysis of FFPE-derived data reliably yields the same results as for FF datasets. For this, we sorted all RefSeq- or ENSEMBL-annotated genes within each experimental group according to their mean expression values and assigned ascending ranks. The ranks of the 100 highest expressed genes in FF arrays were compared with the ranks of these genes in FFPE arrays (Fig. 5g). For rat and human microarrays, more than 55% and 60%, respectively, of the top 100 FF genes (out of >23,000 genes), could be found within the 100 highest expressed genes in FFPE microarrays; 26–29% could be found within a rank difference of 400. Only for less than 16% of the 100 highest expressed genes in rat and human FF arrays, the rank difference was greater than 400. For selected reference and CNS-relevant genes (rat: *Pgk1*, *bAct*, *Atrn*, *Gfap*, *Mbp*, *Mag*, and *Cx3cr1*; human: *G6PD*, *PGK1*, *IL1R1*, *AIF1*, *IL18*, *TLR3*, *HMBS*, *CX3CR1*, *ITGAM and CASP1*), expression levels were additionally determined by qPCR, sorted within each experimental group according to transcript abundance and assigned ascending ranks. For microarray- as well as qPCR-derived data, the ranks of genes in FF datasets were compared with the ranks of the same genes in FFPE datasets (Fig. 5h). The minimal rank differences upon comparison of the selected genes clearly indicated high data concordance between rat and human FF and FFPE microarrays. Only for 1 to 2 genes per comparison, the rank differences were greater than 1. For *Gapdh*-normalized qPCR results, the order of gene expression ranks varied to a higher degree.

For the last comparison of data consistency between FF and FFPE arrays, we changed data normalization to exon level. For each annotated gene with more than one exon, a ratio between the signals derived from the most 3′-end- and most 5′-end-located probe set was calculated (Fig. 5c,d). All FF arrays as well as human FFPE arrays showed a slight tendency for stronger signals at the 3′-end. Rat FFPE arrays showed a minimal trend towards the 5′-end. Increasing tissue lysis time for human samples from 3 to 10 hours did not significantly affect the 3′-end/5′-end ratio (*p* = 0.3587).

Altogether, a broad range of tests to examine data reproducibility and consistency demonstrated a high overall concordance rate within and between the FF and FFPE datasets.

### Gene set enrichment analysis (GSEA) detects similar pathways in FFPE-derived microarrays and fresh tissue-based RNA-Seq datasets

**Figure 6:**
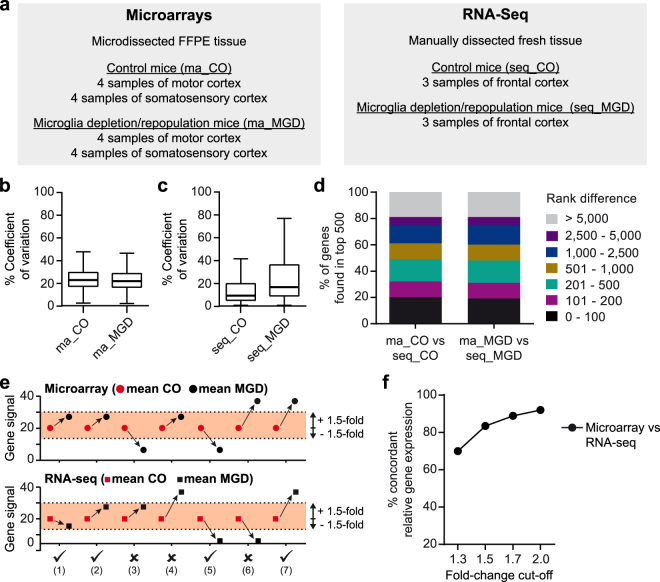
Analysis of data concordance in microarray and RNA-seq datasets. (**a**) Overview of the experimental setup **(b**,**c)** Coefficients of variation (CV, in percent) calculated for control (CO) and microglia depletion/repopulation (MGD) mouse tissue derived from FFPE microarrays (indicated by the prefix “ma”) and fresh tissue RNA-seq (indicated by the prefix “seq”). For each experimental group, the CV for every gene (microarrays: n = 21,520 genes; RNA-seq: n = 16,207 genes) was calculated. (**d**) Genes within each experimental group were sorted and ranked according to their expression rate. Each rank of the 500 highest expressed genes in FFPE microarrays was compared with the corresponding rank in the RNA-seq datasets. The resulting rank differences were categorized as shown. Microarray and RNA-Seq datasets comprised 21,520 and 16,207 genes, respectively. (**e**) Graphical representation of the applied principle for the calculation of concordant relative gene expression between FFPE microarrays and fresh tissue RNA-seq datasets. For each gene in microarray and RNA-Seq datasets, a ratio between the mean value of the MGD group (black dots and squares) and the mean value of the CO group (red dots and squares) was calculated. Concordant relative gene expression (indicated by a tick) was assumed if the two ratios either lay within a defined fold-change cut-off (e.g. ±1.5 as indicated by coloured areas; exemplified by conditions 1 and 2) or showed comparable up- or down-regulation behaviour as depicted by conditions (5) and (7). (**f**) Rate of concordant relative gene expression (in percent) between microarray and RNA-seq datasets for four different fold-change cut-offs calculated as described in (**e**). All datasets comprised 15,270 genes.

For the final confirmation that gene expression profiling with small input amounts of FFPE tissue is reproducibly feasible, we used an experimental mouse model (CX3CR1-Cre^ERT2+/−^/iDTR^+/−^; “MGD mice”) characterized by an inducible depletion of microglia, which is followed by microglia repopulation in a pro-inflammatory milieu. For the present study, we used affected cortical areas from MGD mice at a stage of acute microgliosis. For microarray analysis, RNA from laser-microdissected FFPE sections was processed according to established protocols. Transcriptome profiling from manually dissected fresh tissue was done via RNA-Seq (Fig. 6a). All data analyses were based on annotated genes from microarray and RNA-Seq datasets. Also here, reproducibility and concordance of gene signals were assessed. The median %CV of microarray and RNA-Seq datasets were in a consistently low range (Fig. 6b,c). RNA-Seq data, however, showed a higher degree of variability than microarray data.

In a next step, we sorted all annotated genes of each control and experimental group based on their mean expression value and assigned ascending ranks for the 500 highest expressed genes per group. Subsequently, we compared the ranks between the two control (ma_CO vs seq_CO) and the two experimental (ma_MGD vs seq_MGD) groups and categorized them according to the rank differences (Fig. 6d). Around 50% of the 500 highest expressed genes from the microarray datasets could be detected among the top 500 genes within the RNA-Seq datasets; 20% were even found within a rank difference lower than 100. 25% of the 500 highest expressed genes from microarray datasets, however, were found within rank differences of more than 2,500 in the RNA-seq datasets, which contained a total of >16,000 genes.

Since microarray and RNA-Seq data constitute separate entities, we followed the principle of concordant gene behaviour and evaluated whether the relative expression changes between the mean expression values of the control (CO) and MGD groups were comparable between microarray and RNA-Seq experiments. Concordant relative gene expression was assumed if the ratios between the microarray and the RNA-Seq group were either within a certain fold-change cut-off range (conditions (1) and (2) in Fig. 6e) or showed comparable up- or down-regulation (conditions (5) and (7) in Fig. 6e). Calculations were performed using four different fold-change cut-offs: 1.3, 1.5, 1.7, and 2.0. At a fold-change cut-off of ±1.3, a concordance level of 70% was achieved, which steadily increased to more than 90% at a fold-change cut-off of ±2 (Fig. 6f).

In order to verify that transcriptome profiling with FFPE tissue detects similar differentially regulated pathways compared with fresh tissue, we performed gene set enrichment analysis (GSEA) with the genes differentially expressed between MGD and CO mice. Due to severe microgliosis in MGD mice, we expected inflammatory signalling cascades to be significantly upregulated in MGD mice compared with controls. From both microarray and RNA-Seq datasets (Table 2), 5 out of the 5 most significantly enriched pathways in MGD mice related to inflammatory pathways. Since we compared laser-dissected tissue (FFPE microarrays) containing the most affected areas with cruder manually dissected tissue (fresh tissue RNA-Seq), a higher number of inflammatory pathways was enriched in the FFPE-derived MGD tissue, whereas in the RNA-Seq data, the spatially restricted inflammatory responses were diluted by transcriptional changes of the surrounding tissue.

**Table 2:** Top 20 pathways of microarray and RNA-seq datasets.

Microarray		RNA-Seq	
Pathway name	*p* value	Pathway name	*p* value
***Interferon signalling***	<0.00001	***Interferon signalling***	0.00236
***Interferon alpha/beta signalling***	<0.00001	***Cross-presentation of soluble exogenous antigens (endosomes)***	0.00622
***Cytokine signalling in immune system***	<0.00001	***Interferon gamma signalling***	0.01087
***Immune system***	<0.00001	***Dectin-1-mediated noncanonical NF-kB signalling***	0.01308
***ISG15 antiviral mechanism***	<0.00001	***Interferon alpha/beta signalling***	0.01324
***Antiviral mechanism by IFN-stimulated genes***	<0.00001	Insulin-like growth factor-2 mRNA binding proteins (IGF2BPs/IMPs/VICKZs) bind RNA	0.01384
Transcriptional activation of cell cycle inhibitor p21	0.00010	EPHA-mediated growth cone collapse	0.01661
Transcriptional activation of p53 responsive genes	0.00010	Regulation of gene expression by hypoxia-inducible factor	0.02042
Rho GTPases activate PKNs	0.00016	***CLEC7A (dectin-1) signalling***	0.02486
***Negative regulators of RIG-I/MDA5 signalling***	0.00021	Cellular response to hypoxia	0.02851
***Interferon gamma signalling***	0.00025	Regulation of hypoxia-inducible factor (HIF) by oxygen	0.02851
***Regulation of IFNA signalling***	0.00075	TP53 regulates transcription of genes involved in G1 cell cycle arrest	0.03080
Oxidative stress-induced senescence	0.00137	TP53 regulates transcription of caspase activators and caspases	0.03080
RNA polymerase I promoter opening	0.00153	TFAP2 (AP-2) family regulates transcription of growth factors and their receptors	0.03366
***RIG-I/MDA5-mediated induction of Interferon alpha/beta pathways***	0.00189	Protein-protein interactions at synapses	0.03906
Senescence-associated secretory phenotype (SASP)	0.00189	***Antigen processing/cross presentation***	0.04281
***TRAF3-dependent IRF activation pathway***	0.00196	Interactions of neurexins and neuroligins at synapses	0.04933
DNA methylation	0.00208	Autodegradation of Cdh1 by Cdh1:APC/C	0.05549
TP53 regulates metabolic genes	0.00230	***Regulation of IFNA signalling***	0.05629
Cellular senescence	0.00244	***Cytokine signalling in immune system***	0.05672

Differentially expressed genes between CO and MGD mice were determined for the microarray datasets using restrictive fold-change cut-offs (fold-change either ≤−2 or ≥+2, p-value ≤ 0.05; 182 genes). RNA-seq data were subjected to the DESeq2 algorithm (164 genes). The resulting gene lists were submitted to the Reactome pathway database (www.reactome.org) for pathway analysis. The 20 most over-represented pathways are depicted. Biological pathways implicated in immune reactions are highlighted in bold and italics.

Despite the use of different starting materials and different gene expression profiling technologies, similar differentially regulated biological networks could be detected in a mouse model of microglia depletion/repopulation. Additionally, high levels of data reproducibility and relative gene expression concordance indicated that FFPE-derived microarrays are reliably applicable in transcriptome profiling.

## Discussion

Pathological archives can be vast repositories containing fixed tissues from rare disease cases, from patients with fulminant or exceptional disease courses of high pathological significance, but also biological material harvested prior to nowadays state-of-the-art medication. Especially the latter are of importance if disease mechanisms, which might be blurred due to current therapies, are to be studied^20^. In human pathology, tissue has been and still is routinely fixed and embedded into paraffin to ensure easy storage and longevity. However, particularly in earlier days, tissue processing has often neither been done according to standardized fixation protocols, nor well documented. For such material, *in situ* hybridization targeting ubiquitous mRNAs can serve as rough estimate of RNA quality^12^. However, we have already experienced in previous studies^20,21^ that FFPE tissue blocks with strong *in situ* hybridization signals do not necessarily yield high-quality RNA and thus fail to produce meaningful data in downstream experiments. Therefore, more reliable measures to judge RNA quality prior to costly applications are utterly required. Classical RNA integrity analysis using RIN (or equivalents), as routinely done for fresh and FF samples, cannot be reliably applied to FFPE material^13^ since all RNA species are innately fragmented due to formalin fixation. Instead, the DV200, the percentage of RNA fragments with a length of more than 200 nucleotides, might be a viable alternative^17^. Reportedly, a DV200 greater than 70% indicates high-quality RNA, while RNA samples with values between 50 and 70% are of medium quality and demand the use of higher input volumes for transcriptome analysis^17^. RNA samples with DV200 values below 30% have been suggested to be too degraded for further experiments. In our study, we were able to achieve DV200 values clearly above the proposed threshold for high-quality RNA despite very low tissue input. The DV200 was also used as a readout of RNA quality in a recent technical RNA isolation study involving FFPE-derived RNA^14^. In that study, the High Pure FFPE kit (Roche), which was also used by us, led to the highest yields and DV200 values for rat and human renal tissue. For RNA sequencing, these samples generated the greatest amount of total reads as well as reads that could be mapped to the transcriptome. Other isolation kits producing lower RNA yields and DV200 values accordingly performed worse during RNA sequencing. Correspondingly, we consider the DV200 as an objective determinant for RNA quality check-up that mirrors FFPE RNA quality more precisely than the frequently (mis)used RINs. Nevertheless, the visual inspection of RNA electropherograms should not be disregarded, since alterations, like indications of putatively cross-linked nucleic acids might be overlooked. Moreover, our experience in RNA extraction from FFPE tissues has shown that samples with a high DV200 value do not consistently yield detectable amplification products. Instead, qPCR has proven to be a valuable tool for testing the functional applicability in microarray analyses. For qPCR using FFPE-derived templates, amplicon size should be as short as possible^22,23^. Upon comparison of FF and FFPE samples, we and others^24^ have observed that Cq values for FFPE samples are several units higher than those derived from paired FF counterparts. With these peculiarities of FFPE-derived RNA in mind, we highly recommend functional testing of each sample using reference genes along with Bioanalyzer check-up prior to any kind of costly transcriptome profiling.

Most RNA isolation kits for FFPE tissues include a digestion step with proteinase K during tissue lysis, as this was shown to be inevitable for retrieving RNA from FFPE tissue^25,26^. Generally, long incubation times were reported to improve yield and quality^13^. A peak after the usual plateau phase of RNA electropherograms has been described to indicate residual, cross-linked nucleic acids^18,19^. In our case, using biopsy tissue that was optimally fixed (24 hours only), an extension of the lysis time from 3 (as suggested by the manufacturer) to 10 hours reduced the amount of high-molecular-weight species representing putatively cross-linked nucleic acids; however, it had no effects (neither beneficial nor detrimental) on RNA quality parameters and functional applicability. Nevertheless, in case of FFPE autopsy tissue as starting material, for which fixation protocols are often not standardized, an increase in tissue lysis time might positively influence performance parameters. Although we cannot fully exclude that prolonging the lysis time might increase RNA fragmentation, the latter did not appear to be a significant factor in our experiments as indicated by the stable RNA quality parameters in our data.

Prolonged tissue lysis does not reverse chemical modifications of the RNA. For this, an additional improvement of the original tissue lysis protocol was made, namely an extra incubation step at 70 °C. It has been described that at this temperature, the majority of chemical modifications is released from nucleic acids, while the phosphate backbone is not hydrolysed^25^. This additional incubation step also drastically increased the amount of retrieved RNA, which is crucial, since the quantities of precious tissue available for transcriptome profiling are often limited and thus, there is an increasing need for reliable and efficient generation of gene expression data based on sparse starting material. From as little as 2 ng of total RNA, we were able to receive whole-genome microarray data that fulfilled all internal quality criteria and were comparable to FF-derived data in a broad range of correlations, concordance tests and 3′-end/5′-end ratio analysis. We did not test lower input amounts for this study, yet, the minimum input limits of the employed kit are even lower (100 pg and 500 pg for FF and FFPE tissue, respectively). Similarly as for cDNA preparation for qPCR, this kit contained not only oligo(dT) primers but also random hexamers to allow for transcription of as many RNA fragments as possible. Moreover, a technical bias towards 3′-end poly(A) tails was avoided. We chose the Affymetrix GeneChip^TM^ technology for gene expression profiling since microarray technology has greatly advanced and offers a cost-efficient, robust and easy-to-analyse profiling platform. Most genes are covered by several probes per exon, providing a comprehensive image of the transcriptome without introducing a 3′-end bias. Generally, the 3′-end poly(A) tail was shown to be strongly prone to chemical modification since adenine is the base most susceptible to fixation-induced alterations^25^, thus possibly rendering it less accessible for poly(T) primers.

Reproducibility and consistency of paired FF- and FFPE-derived datasets can be assessed using several measures. In comparison with other studies^15,27,28^, the Pearson’s correlation coefficients obtained by us for FF or FFPE replicates as well as FF-FFPE pairs were in the same range or even higher. For example, Roberts *et al*. reported coefficients in the range of 0.89 to 0.99 for intragroup replicates (FF versus FF; FFPE versus FFPE) and slightly lower coefficients of 0.82 to 0.89 for paired FF and FFPE samples^15^. Pearson’s correlation coefficients between FF-FFPE pairs using RNA sequencing technologies also fall into this range^29^. A major difference, however, is the much lower input amount in our study.

FF- and FFPE-derived microarrays yield inherently differing data entities and thus, absolute gene expression signals should not be directly compared with each other. Therefore, we used relative indirect measures for our analyses. In the MicroArray Quality Control project (MAQC)^30^, intraplatform data reproducibility was reviewed by the CV, which is a quality control measure accounting for the innately increased variation of highly expressed genes. Using universal reference RNAs, the median CV values of Affymetrix microarrays were between 5 and 10% in the MAQC study. Our results are clearly above this range; however, our microdissected samples are based on FFPE material, which is naturally inferior in quality for RNA studies. Some other microarray platforms tested in the MAQC projects either came close to or were in the range of the CV values observed in our study.

Eikrem and collaborators recently compared the 20 most up- or downregulated genes in FF- and FFPE-based datasets derived from RNA sequencing of renal carcinoma specimen^31^. They detected 70% of the top 20 genes from the FF list also within the FFPE counterpart and vice versa. The remaining 30% of genes were either ranked higher than 20 or could not be found at all in the corresponding gene lists. We performed rather similar analyses; however, we included either the top 100 (for microarray comparison between FF and FFPE) or 500 (for the comparison between microarray and RNA-Seq datasets) highest expressed genes. Our detection rates of 50 to 60% were lower than the ones observed by Eikrem and colleagues. However, they based their analyses on pre-sorted gene lists that included only p-value-filtered ENSEMBL-annotated genes, while we employed a broader, unsupervised dataset, as commonly used as basis for the detection of differentially expressed genes. In an additional small-scale analysis, rank differences of selected major reference and CNS-relevant genes derived from the FF and FFPE microarray datasets were only negligible, thus indicating high data concordance. For qPCR results, gene expression ranks varied to a higher degree, which we hypothesize to be due to differing template preparations and normalization methods. For microarray hybridization, transcripts were amplified for several rounds, while cDNA for qPCR was reversely transcribed directly from the isolated RNA. Thus, detection of low abundance transcripts might vary to a higher degree for qPCR. Moreover, normalization of qPCR results commonly bases on selected reference genes, while microarray normalization is more robust due to normalization across all microarray signals.

In order to evaluate further performance measures between FF and FFPE microarrays, relative expression changes between the biological replicates can be calculated for the different experimental groups. If tissue handling (e.g. fixation, embedding, storage, dissection) does indeed not impact data reproducibility and consistency, the relation of the gene signals should be maintained between FF and FFPE arrays. In gene expression studies, genes, which are significantly up- or downregulated above a fold-change level of 2, are usually considered as differentially expressed. Using this cut-off range in our analysis of concordant relative gene expression, we could show that around 90% of all analysed RefSeq- or ENSEMBL-annotated genes maintained their relative expression in FFPE microarrays as compared with FF material-derived counterparts underlining the trustworthiness of FFPE results. The reliability of FFPE microarrays was further strengthened by applying the same relative gene expression calculations on the microarray and RNA-Seq datasets derived from the experimental mouse model. Also here, we could show that up to 90% of the annotated genes have a similar up- or down-regulation behaviour.

Altogether, our study shows that FFPE-derived microarrays can be analysed similarly as FF-derived arrays and generate reliable and reproducible datasets. Particularly, pathway analysis via GSEA in well-preserved FFPE tissues led to the detection of similar biological pathways compared with fresh tissue-based datasets. We may point out that our workflow for FFPE tissue-based microarrays was specifically designed for the analysis of global gene expression changes, e.g. differentially expressed pathways. Data variation in FFPE arrays was higher when reviewed on single-gene basis, which constitutes a notable limitation of the study. We therefore suggest to refrain from comparisons of single-gene expression rates and recommend whole-genome analyses instead. Other in-depth RNA analyses such as splice variant analysis, microRNA profiling or SNP analysis, were not targeted by our study.

Certainly, the use of FFPE tissues for microarray analysis has its limitations. Importantly, the RNA quality of these biological materials can markedly differ in the type of tissue harvest (biopsy, autopsy, perfused experimental tissues) and storage conditions with concomitant negative influences such as increased autolysis and fixation times^8,10,11,13^. Most importantly, formalin-caused nucleotide modifications can strongly vary between individual specimens, thereby interfering with downstream applications and leading to larger data variation within experimental groups. In order to achieve the best RNA quality and yield, we thus strongly recommend optimizing RNA isolation steps such as tissue lysis time and additional heating. Independent of the choice of starting material, the use of stringent statistical tools considering false discovery rate (FDR) and false negative rate (FNR)^32^ is encouraged for the analysis of transcriptome data. Additionally, we suggest independent gene level-based (qPCR, *in situ* hybridization, etc.) and protein level-based (immunohistochemistry, western blot, etc.) verification of selected molecules/pathways of interest. Moreover, we propose to refrain from the comparison of absolute gene expression data from FF and FFPE material in a joint analysis, since these constitute inherently differing data entities. For the comparison of performance parameters (e.g. in the scope of technical studies), indirect measures, as used by us, can be employed.

Despite some limitations, our study provides solid evidence for the usability of FFPE microarrays in pathway-wide and hypothesis-generating transcriptome profiling. The described workflow of RNA isolation, quality assessment and microarray preparation can be employed even with minimal quantities of starting material as often required when handling precious but pathologically highly significant human tissue. Our study consolidates the usability of FFPE tissues collected world-wide within archives and tissue banks over decades and encourages research not feasible with FF tissue, thereby opening new doors in human molecular pathology and the search for disease-driving candidate pathways.

## Materials and Methods

### Tissue sources

Wild‐type Lewis rats were bred and housed under standardized conditions in the Decentral Facilities of the Institute of Biomedical Research (Medical University of Vienna) with access to food and water ad libitum.

CX3CR1-Cre^ERT2+/−^/iDTR^+/−^ mice were bred and conventionally housed and treated in specific-pathogen-free conditions at the Harvard Institutes of Medicine according to the animal protocol with full knowledge and permission of the Standing Committee on Animals at Harvard Medical School. Mice were injected i.p. with 10 µg of tamoxifen (Sigma-Aldrich) dissolved in peanut oil for 5 consecutive days. Microglia depletion was induced by injection of 1 µg diphtheria toxin (Sigma-Aldrich) per mouse for three consecutive days. Mice receiving tamoxifen but not any diphtheria toxin injections were used for control reasons.

Human tissue samples were acquired in 2014 and 2015 from surgical material (right hemisphere) of three epileptic patients suffering from Rasmussen encephalitis, in the Epilepsy Center Bethel (Bielefeld, Germany). For the two male patients, surgical intervention was done at the age of 4.9 and 6.8 years. The female subject was 26.9 years old. The procedures involving human participants were approved by the regional ethical committee at the University of Münster, Germany (2015-088-fs). The participants or their legal representatives had given informed consent. The study conformed with the Helsinki declaration (1964) and its amendments or comparable ethical standards.

### Tissue processing

For protocols treating rat tissue, xylene, ethanol (EtOH) and staining solution were derived from the Histogene® LCM Frozen Section Staining Kit (Cat.No. KIT0401, Thermo Fisher Scientific), while for all other protocols the following materials were used: xylene (28975.325, VWR), EtOH absolute (100983, Merck Millipore), 2-propanol (109634, Merck Millipore). To adsorb water from 100% EtOH, 2-propanol and xylene, molecular sieves (Type 4A, Cat.No. 32256; Alfa Aesar) were added to the staining jars. All glassware was baked for 4 hours at 180 °C to eliminate RNase activity. Tissues for FFPE processing were fixed in 4% paraformaldehyde (PFA) in 0.1 M Soerensen phosphate buffer (pH 7.4). Routine tissue dehydration and paraffination was done in a Tissue-Tek VIP-2000 tissue processor (Sakura) and included the following consecutive incubations: 50% EtOH for 20 minutes (40 °C), twice 70% EtOH for 1 hour and 1.5 hours (40 °C), twice 80% EtOH for 1 hour and 1.5 hours (40 °C), three times 96% EtOH for 1, 1.5 and 2 hours (40 °C),twice xylene for 30 minutes and 1 hour (40 °C) and finally three times paraffin each for 1 hour at 60 °C (Histosec® paraffin pastilles, Cat.No. 111609, Merck Millipore). At the end, tissues were placed in embedding cassettes and subsequently, paraffin blocks were stored in the archives of the Center for Brain Research.

#### Fresh tissue from mice

Mice were sacrificed by CO_2_ asphyxiation and transcardially perfused with 20 ml of ice-cold HBSS. Brains were removed and the frontal cortex was manually dissected and stored in Trizol at −80 °C.

#### FFPE tissue from mice

Mice were sacrificed by CO_2_ asphyxiation and transcardially perfused with 20 ml of ice-cold HBSS. Brains were dissected and fixed in 4% PFA overnight at 4 °C. The next day, the tissue was transferred to PBS. Coronal brain sections were cut and the tissue was routinely dehydrated and paraffinized as described before. 8 µm sections were cut using a microtome and mounted on RNase- and nucleic acid-free polyethylene terephthalate (PET) membrane slides (Cat.No. 50102; Molecular Machines & Industries, MMI), which were further stored for at least 2 days at room temperature in RNase-free slide boxes filled with desiccant (Drierite® 8 mesh, Cat.No. 89751; Alfa Aesar). Slides were deparaffinized twice in xylene (each for 2 minutes) and washed in 2-propanol for 45 seconds and nuclease-free water (Cat.No. 10977035; Thermo Fisher Scientific) for 30 seconds. Tissue sections were incubated for 30 seconds with hematoxylin stain (H&E Staining Kit Plus, MMI). The solution was decanted and the slides were washed twice in nuclease-free water (each for 30 seconds) and once in 2-propanol for 45 seconds. After 4 minutes incubation in xylene, the membrane slides were air-dried for 1 minute and immediately used for microdissection.

#### FF tissue from rats

Wild-type Lewis rats (male; 4 months old) were sacrificed by CO_2_ asphyxiation and transcardially perfused with 60 ml PBS. Brains were dissected and coronally cut into 2–3 mm thick sections, which were immediately snap-frozen on pre-cooled RNase-free plastic dishes placed in liquid N_2_. Tissue sections were subsequently stored at −80 °C. Prior to cryostat cutting, the tissue was kept at −20 °C for 20 minutes to reach optimal cutting temperature. Subsequently, 16 µm sections were cut and mounted on RNase- and nucleic acid-free PET membrane slides, which were stored at −80 °C. Slides were allowed to defrost for 20 seconds before incubation in ice-cold 75% EtOH and nuclease-free water each for 30 seconds. Thereafter, tissue sections were incubated for 30 seconds with 100 µl staining solution supplemented with 1 µl RiboLock RNase Inhibitor (Cat.No. EO0381; Thermo Fisher Scientific). The solution was decanted and slides were washed and dehydrated in a series of nuclease-free water, 75% EtOH, 95% EtOH (each for 30 seconds), and 100% EtOH for 3 minutes. After 5 minutes incubation in xylene, membrane slides were air-dried for 2 minutes and immediately used for microdissection.

#### FFPE tissue from rats

Wild-type Lewis rats (male; 4 months old) were sacrificed by CO_2_ asphyxiation and transcardially perfused with 60 ml 4% PFA. Brains were dissected and post-fixed in 4% PFA for 24 hours at 4 °C. After thorough washing in PBS, coronal sections were cut and the tissue was routinely dehydrated and paraffinized as described above. Paraffin blocks were stored at 4 °C. 8 µm sections were cut using a microtome and mounted on RNase- and nucleic acid-free PET membrane slides, which were further stored for at least 2 days at room temperature in RNase-free slide boxes filled with desiccant. Slides were deparaffinized in xylene (2 × 2 minutes) and rehydrated in 100% EtOH for 2 minutes followed by 95% EtOH and 75% EtOH for 1 minute each, and nuclease-free water for 30 seconds. Thereafter, tissue sections were incubated for 30 seconds with 100 µl staining solution supplemented with 1 µl RiboLock RNase Inhibitor. The solution was decanted and the slides were washed and dehydrated in a series of 75% EtOH and 95% EtOH (each for 30 seconds) and 100% EtOH for 3 minutes. After 5 minutes incubation in xylene, the membrane slides were air-dried for 2 minutes and immediately used for microdissection.

#### Human tissue

Immediately after surgical resection of hippocampi, they were coronally cut into two parts with an approximate size of 0.5 × 2 cm. One part was immediately snap-frozen in liquid N_2_ and stored at −80 °C. The second half was fixed in 4% PFA for 24 hours at 4 °C, thoroughly washed in PBS and embedded in paraffin as described above. Paraffin blocks were stored at room temperature.

#### Human FF tissue

Prior to cryostat cutting, tissue was kept at −20 °C for 20 minutes to reach optimal cutting temperature. Per sample, two consecutive 16 µm sections were cut and mounted on RNase- and nucleic acid-free glass slides (Superfrost Plus; Thermo Fisher Scientific). One slide was allowed to air-dry at room temperature; the other slide was stored at −80 °C. The dried slide was routinely stained with hematoxylin and eosin (H&E) and the hippocampus was marked as a template for subsequent RNA isolation.

#### Human FFPE tissue

Per sample, three 8 µm sections were cut and mounted on RNase- and nucleic acid-free glass slides. Two slides were stored at room temperature for at least 2 days in an RNase-free slide box filled with desiccant. The third slide was routinely stained with H&E and the hippocampus was marked as a template for subsequent RNA isolation.

### Dissection of regions of interest

#### Mice and rats (FF and FFPE tissue)

Air-dried membrane slides were processed within 40 minutes of completion of staining/dehydration. Microdissection was carried out using an inverted light microscope (Nikon Eclipse Ti) equipped with an MMI CellCut laser microdissection system. Using CellTools v4.4 software (MMI), regions of interest were drawn and cut out using a UV solid state laser. Specimen were collected on isolation caps (Cat.No. 50202; MMI) and covered with lysis buffer as soon as sufficient amounts of tissue were harvested.

#### Human FF tissue

Each section was thawed at room temperature for 30 seconds and the hippocampus was macroscopically dissected using the H&E-stained slide as template. Tissue was collected in a tube and 50 µl extraction buffer was immediately added.

#### Human FFPE tissue

Hippocampi were macroscopically dissected using the H&E-stained slide as template and collected in a tube. All subsequent deparaffination steps were performed at room temperature. First, 800 µl xylene was added to the samples, which were thoroughly vortexed, followed by 2 minutes incubation. Samples were once again vortexed and incubated with xylene for 5 minutes. Tubes were centrifuged at 13,000 × *g* for 2 minutes and the supernatant was aspirated. This step was repeated once. Then, 800 µl 100% EtOH was added and the samples were vortexed, followed by centrifugation and aspiration of 100% EtOH. This step was repeated once with 70% EtOH. Thereafter, samples were centrifuged for 30 seconds to remove EtOH. Tissue pellets were dried in an RNase-free box at 55 °C and covered with 70 µl lysis buffer.

### Tissue lysis and RNA isolation

#### Fresh tissue

RNA was isolated using Trizol extraction as previously described^33^.

#### FF tissue

Using the PicoPure® RNA Isolation Kit (Cat.No. KIT0204; Thermo Fisher Scientific) according to manufacturer’s instructions, the dissected rat and human tissues were lysed for 30 minutes at 42 °C and RNA was eluted in 11 µl elution buffer, aliquoted and stored at −80 °C.

#### FFPE tissue

For RNA isolation from FFPE material, the High Pure FFPE RNA Micro Kit (Cat.No. 04823125001; Roche) was used. Microdissected rat tissue was lysed for 3 hours at 55 °C in lysis buffer supplemented with proteinase K (included in the isolation kit) and 10% sodium dodecyl sulfate (SDS). Microdissected mouse tissue was lysed for 3 hours at 55 °C in supplemented lysis buffer followed by incubation at 70 °C for 20 min. Human tissue was lysed for either 3 or 10 hours at 55 °C in supplemented lysis buffer followed by incubation at 70 °C for 20 min. For all samples, RNA was eluted in 20 µl elution buffer, aliquoted and stored at −80 °C.

### Agilent 2100 Bioanalyzer

RNA integrity of samples isolated from fresh mouse tissue was determined by Agilent 2100 Bioanalyzer using Agilent RNA Nano Chips. Samples with an RNA integrity score (RIN) higher than 8 qualified for further processing. To determine RNA integrity and quantity of FF and FFPE samples, 1 µl total RNA was loaded on Agilent RNA Pico Chips. For each sample, the electropherogram was plotted and RIN and DV200 were calculated using 2100 Expert software (version B.02.08.SI648 (SR2)).

### Reverse transcription (RT) and qPCR

For qPCR analysis of FF as well as FFPE samples, 4 ng total RNA was transcribed into cDNA using the iScript^TM^ cDNA Synthesis Kit (Cat.No. 1708890; Bio-Rad) according to the manufacturer’s instructions. Per qPCR reaction, cDNA template (200 pg RNA equivalents) was mixed with SsoAdvanced^TM^ Universal SYBR® Green Supermix (Cat.No. 1725270; Bio-Rad), forward and reverse primers (final concentration: 200 nM per primer) and nuclease-free water to a volume of 10 µl. The following thermal cycling conditions were applied on a StepOnePlus^TM^ Real-Time PCR System (Applied Biosystems^TM^, Thermo Fisher Scientific): 30 seconds at 95 °C; 40 cycles alternating 15 seconds at 95 °C and 1 minute at 60 °C. Subsequently, routine melting curve analysis was conducted. Data were analysed with ExpressionSuite Software v1.0.3 (Applied Biosystems^TM^, Thermo Fisher Scientific). Employed primers are listed in Table 1. All primer pairs were tested for efficiency. For functional RNA quality assessment, three reference genes per species (rat: glyceraldehyde-3-phosphate dehydrogenase (*Gapdh*), phosphoglycerate kinase 1 (*Pgk1*) and actin beta (*bAct)*; human: *GAPDH*, glucose-6-phosphate dehydrogenase (*G6PD*) and succinate dehydrogenase complex flavoprotein subunit A (*SDHA*)) were selected and two different primer sets were designed for each gene: one annealing near the 5′-end of the mRNA and the other pair targeting a sequence near the 3′-end. For validation of microarray data, the following genes were analysed: (i) rat: *Gapdh*, *Pgk1*, *bAct*, attractin (*Atrn*), glial fibrillary acidic protein (*Gfap*), myelin basic protein (*Mbp*), myelin-associated glycoprotein (*Mag*) and chemokine (C-X3-C motif) receptor 1 (*Cx3cr1*); (ii) human: *GAPDH*, *PGK1*, *CX3CR1*, glucose-6-phosphate dehydrogenase (*G6PD*), interleukin 1 receptor, type I (*IL1R1*), allograft inflammatory factor 1 (*AIF1*), interleukin 18 (*IL18*), toll-like receptor 3 (*TLR3*), hydroxymethylbilane synthase (*HMBS*), integrin alpha M (*ITGAM*) and caspase 1 (CASP1). CQ values of genes of interest (GOI) were normalized to *Gapdh* (ΔCQ = CQ^Gapdh^ − CQ^GOI^) and subsequently ranked according to transcript abundance (highest expressed gene was assigned rank 1, etc.).

### Affymetrix GeneChip^TM^ whole-genome microarrays

For all FF- and FFPE-derived samples from rat, mouse and human origin, 2 ng total RNA were used as input for cDNA preparation using the GeneChip^TM^ WT Pico Kit (Cat.No. 902623; Affymetrix, Thermo Fisher Scientific). A total of 9 cycles pre-IVT (*in vitro* transcription) amplification was run. Fragmented and labelled samples were hybridized to either GeneChip^TM^ Human Gene 2.0 ST Arrays, GeneChip^TM^ Rat Gene 2.0 ST Arrays or GeneChip^TM^ Mouse Gene 2.1 ST 16-Array Plate (all Affymetrix, Thermo Fisher Scientific). Scanning was done on a GeneChip^TM^ Scanner 3000 7G or GeneTitan^TM^ MC Instrument (both Affymetrix, Thermo Fisher Scientific). The resulting CEL files were loaded into Affymetrix Expression Console^TM^ software (v1.4.1). Per species, FF and FFPE arrays were normalized together applying the RMA-Sketch algorithm. Quality metric reports and normalized datasets were exported from the EC software. Microarray data were deposited in NCBI’s Gene Expression Omnibus (GSE104568 and GSE104634).

### RNA-Sequencing (RNA-Seq)

RNA quantity was determined by Nanodrop measurement. Samples were adjusted to a minimum of 5 ng/µl and 250 ng in total. Libraries were constructed using Illumina’s TruSeq kit with poly(A) selection, pooled and sequenced on the Illumina NextSEQ with 75 bp paired-end reads to a read coverage of 30 million reads per sample. RNA-Seq reads were aligned using Tophat^34^ and RSEM-based quantification based on known transcripts^35^. Further processing was done using the Bioconductor package DESeq in R^36^. Data was normalized via TMM normalization^35^ and post-processing and statistical analysis was carried out in R^35^. Differentially expressed genes were defined using the differential expression pipeline on the raw counts with a single call to the function DESeq^36^ (adjusted p value < 0.1).

### Data analysis and statistics

Significance testing, Pearson’s correlations and graphical data representations were done using SPSS® Statistics v21 (IBM) and GraphPad Prism® v6.01. Box plots indicate the median value and the upper and lower quartiles. Whiskers were drawn according to Tukey’s method and outliers were not depicted. Unsupervised hierarchical clustering (Pearson’s correlation; average linkage) was based on Log2-transformed gene signals and conducted in Multiple Experiment Viewer (MeV) v4.8.1^37^. For Pearson’s correlation coefficients, Log2-transformed datasets were used as well. Statistical tests for coefficients of variation (ratio of the standard deviation to the mean) were based on root-of-three-transformed data. Generally, significance levels were calculated using unpaired, two-tailed Student’s t-tests. Effect sizes for significant differences in large sample sets were determined via Cohen’s *d*. The macro for the transcriptome-wide calculation of 3′-end/5′-end ratios was written in Visual Basic for Applications v7.0 and run in Excel 2010 (both Microsoft). For gene set enrichment analysis (GSEA) of microarray data, differentially expressed genes based on 2-fold up- or down-regulation, which was below a significance level of 0.05. Gene lists were submitted to the reactome pathway database^38,39^ for GSEA.

For the analysis of concordant relative gene expression between FF and FFPE microarrays, gene expression ratios (black and grey arrows in Fig. 5e) between the three biological replicates (red, black and grey dots and squares in Fig. 5e) were calculated for each experimental group (r_FF, r_FFPE, h_FF, h_FFPE_3 h and h_FFPE_10 h). Concordant relative gene expression (indicated by a tick in Fig. 5e) was assigned if the gene expression ratios of the two compared experimental groups (e.g. r_FF compared with r_FFPE) fulfilled one of following requirements: (i) both gene expression ratios were within a defined fold-change cut-off (representing supposedly unchanged gene expression; e.g. cut-off of 1.5 as represented in Fig. 5e) independent of any up- or down-regulation of gene expression (variants (1) and (2) in Fig. 5e); (ii) the ratios were higher/lower than the defined fold-change cut-off but the up-/down-regulation behaviour was the same for FF and FFPE replicates (variants (3) and (5) in Fig. 5e). The latter was not the case, if gene expression ratios between the biological replicates did not fully match between the investigated experimental groups (variants (4) and (6) in Fig. 5e). For each investigated fold-change cut-off (1.3, 1.5, 1.7 and 2.0), gene expression ratios were calculated three times for each of the RefSeq- or ENSEMBL-annotated genes with every biological replicate (red, black and grey dots and squares in Fig. 5e) serving as basis once. For the analysis of concordant relative gene expression between FFPE microarrays and fresh tissue RNA-Seq, expression ratios between the mean value of the MGD group (black dot or square in Fig. 6e) and the mean value of the CO group (red dot or square in Fig. 6e) were calculated for each of the analysed genes for four different fold-change cut-offs (1.3, 1.5, 1.7 and 2.0). Also here, concordant relative gene expression was assumed if the two ratios (derived from the microarrays and the RNA-seq study) either lay within a defined fold-change cut-off (variants (1) and (2) in Fig. 6e) or showed the same up- or down-regulation behaviour (variants (5) and (7) in Fig. 6e).
